# Functional role of the Tau protein in epithelial ovarian cancer cells

**DOI:** 10.1002/rmb2.12019

**Published:** 2017-03-20

**Authors:** Aisa Yamauchi, Asami Kobayashi, Hiroe Oikiri, Yoshihito Yokoyama

**Affiliations:** ^1^ Department of Obstetrics and Gynecology Hirosaki University Graduate School of Medicine Hirosaki Japan

**Keywords:** apoptosis, cell proliferation, **e**pithelial ovarian cancer, paclitaxel, Tau protein

## Abstract

**Aim:**

The microtubule‐associated Tau protein is a marker of paclitaxel sensitivity in ovarian cancer. The aim of the present study was to elucidate the function of the Tau protein in epithelial ovarian cancer.

**Methods:**

The correlation between Tau protein expression and the response to paclitaxel by using several ovarian cancer cell lines was investigated.

**Results:**

A Western blot showed that the expression level of the Tau protein was the highest in the TOV112D cells. A cell‐counting kit showed that the proliferation rates were more inhibited in the cells with down‐regulated Tau protein than in the control cells, both with and without paclitaxel treatment. The proliferation rates of the control cells and the TOV112D cells also were compared with Tau protein overexpression. The level of cell proliferation was more inhibited in the cells that overexpressed the Tau protein, compared to the control cells, both with and without paclitaxel treatment. It was shown that both the down‐regulation and the overexpression of the Tau protein were related to the inhibition of TOV112D cell proliferation. Early and late apoptosis of the TOV112D cells that were transfected with Tau cDNA plasmid construct or Tau small interfering RNA significantly increased.

**Conclusion:**

These findings suggest that the molecular targeting of the Tau protein could be a potential treatment for ovarian cancer.

## 1 Introduction

Ovarian cancer (OC) is the leading cause of death among patients with gynecological cancer. Paclitaxel and platinum‐based chemotherapy often are used as the first‐line chemotherapy in the treatment of patients with epithelial OC. Almost all patients with epithelial OC are good responders to paclitaxel and platinum‐based chemotherapy. However, some patients experience a relapse and require second‐line chemotherapy. A useful marker of a sensitivity to paclitaxel and platinum‐based chemotherapy has not been previously identified.

It is well known that the Tau protein is related to Alzheimers disease. The accumulation of hyperphosphorylated and aggregated microtubule‐associated protein Tau (MAPT) is a central feature of a class of neurodegenerative diseases that are termed “tauopathies.” The continuing increase in the adult‐born neurons indicates that tauopathies are characterized by increased neurogenesis.^1^ The molecular weight of the Tau protein is 50‐64 kDa and it is a product of the *TAU* gene that is located in chromosome 17. In addition to neurons, Tau is expressed at low levels in several non‐neuronal cells. The Tau protein consists of a N‐terminus and a C‐terminus. It has a microtubule‐binding part at the C‐terminus. In the mammalian brain, the Tau protein has six isoforms that differ in the number of microtubule‐binding domain repeats (three or four).^2^ The Tau protein binds to beta‐tubulin and thus stabilizes the microtubules. Paclitaxel exhibits its effect through the exact same mechanism. Therefore, the Tau protein and paclitaxel compete for binding to the microtubules.

Recently, the Tau protein has been identified as a marker of response to paclitaxel in breast cancer and OC.^3^, ^4^ It was reported that the negative expression of the Tau protein appears to be both a good prognostic factor and a predictor of the response to paclitaxel and platinum‐based chemotherapy in patients with epithelial OC.^4^ Another study also reported that low Tau protein expression might be used as a marker to select patients for paclitaxel therapy.^5^ In the field of gynecological oncology, very little work has been done on the Tau protein. As described above, the Tau protein and paclitaxel bind to the same part of the microtubules, thereby promoting tubulin polymerization and microtubule stabilization. This is the mechanism of the antitumor action of paclitaxel. In the field of neurology, it is well known that phosphorylation of the Tau protein causes apoptosis of the neurons; the Tau protein is closely related with cell apoptosis. Therefore, the authors researched the function of the Tau protein with respect to the effect of paclitaxel. The OC cell line was selected because paclitaxel and platinum‐based chemotherapy are the first‐line chemotherapy regimen for epithelial OC. However, the role of the Tau protein in cancer cells has not been clarified yet. Therefore, Tau protein function was analyzed in epithelial OC cells in this study.

## 2 Materials and Methods

### 2.1 Cell lines and cell culture

The TOV112D, MCAS, and OVCAR3 cell lines were obtained from the American Type Culture Collection (Rockville, MD, USA). The TOV112D is derived from human endometrioid carcinoma,^6^ MCAS is derived from human mucinous carcinoma,^7^ and OVCAR‐3 is derived from human serous carcinoma.^8^ The OVICE cell line that is derived from human clear cell carcinoma was obtained from the Japanese Collection of Research Bioresources Cell Bank (Osaka, Japan).^9^ The HRA cells and DISS cells, derived from human epithelial ovarian carcinoma, were generously provided by the National Defense Medical College, Tokorozawa, Japan,^10^ and Jichi Medical School, Tochigi, Japan^11^, respectively. All the cells were grown in Roswell Park Memorial Institute (RPMI) medium‐1640 (Sigma‐Aldrich, St. Louis, MO, USA) and supplemented with 10% fetal bovine serum, at 37°C, in a water‐saturated atmosphere with 5% CO_2_ and 95% air.^12^ All the cell lines were verified in writing as being ovarian in origin and no mycoplasma contamination was present.^12^

### 2.2 Plasmid DNA preparation

A pCMV6‐AC‐GFP vector (OriGene Technologies, Inc., Rockville, MD, USA) was used that encodes the human MAPT transcript variant 1 and is fused to green fluorescent protein (GFP) and the ampicillin resistance gene. For amplification, pCMV6‐AC‐GFP was transformed into DH5α‐competent cells via heat‐shock transformation, according to the standard laboratory protocols.^13^ The transformed bacteria were amplified in lysogeny broth–ampicillin medium.^13^ The plasmids were purified from cultured, transformed bacteria by using a PureLink HiPure Plasmid Filter Maxiprep DNA purification kit (Invitrogen Life Technologies, Carlsbad, CA, USA), according to the manufacturer's instructions.^13^ The plasmid DNA was diluted in sterile water at a concentration of 3 μg/μL.^13^

### 2.3 Small interfering RNA preparation

The sequence of the small interfering RNA (siRNA) duplex that was specific for MAPT was synthesized commercially by OriGene Technologies. The siRNA tube was briefly centrifuged in order to ensure that all the contents collected at the bottom of the tube and the duplexes were resuspended in the provided RNase‐free duplex buffer. The tube was heated to 94°C for 2 minutes and subsequently cooled to room temperature.

### 2.4 In vitro plasmid DNA transfection

One day before transfection, 0.5×10^7^ TOV112D and HRA cells were plated and cultured in 15 mL of RPMI medium‐1640 that was supplemented with 10% fetal bovine serum without antibiotics in 10 cm culture dishes so that they would reach 80% confluence at transfection.^12^ Lipofectamine 3000 (Thermo Fisher Scientific, Waltham, MA, USA) was used for the DNA transfection. Next, 24 μg of MAPT DNA was diluted in 1000 μL of phosphate‐buffered saline (PBS) and 60 μL of lipofectamine was diluted in 1000 μL of PBS. Then, the dilutions were incubated for 5 minutes at room temperature. After the incubation, the DNA and lipofectamine were combined and incubated for 20 minutes at room temperature. After the incubation, 2000 μL of the complexes were added to each 10 cm culture dish. The vector without MAPT DNA was used as the control. The control cells were given only a mixture of the vector and lipofectamine.

### 2.5 In vitro small interfering RNA transfection

One day before transfection, 0.2×10^7^ TOV112D and OVCAR3 cells were plated and cultured in 15 mL of RPMI medium‐1640 that was supplemented with 10% fetal bovine serum without antibiotics in 10 cm culture dishes so that they would reach 50% confluence at transfection.^12^ Lipofectamine 3000 was used for the siRNA transfection. First, 600 pmol of MAPT siRNA was diluted in 1000 μL of PBS and 30 μL of lipofectamine in 1000 μL of PBS. Then, the dilutions were incubated for 5 minutes at room temperature. Next, the diluted siRNA was combined with the diluted lipofectamine and incubated for 20 minutes at room temperature. After the incubation, 2000 μL of the complexes were added to each 10 cm culture dish. Control siRNA‐A (sc‐36869; Santa Cruz Biotechnology, Santa Cruz, CA, USA), instead of MAPT siRNA, served as a control.

### 2.6 Western blot analysis

Cell lysates were prepared from the TOV112D, OVCAR3, HRA, DISS, OVISE, and MCAS cells. In addition, cell lysates were prepared from the TOV112D and HRA cells that had been cultured with the solution of lipofectamine and MAPT DNA complex for 48 hours and from the TOV112D and OVCAR3 cells that had been cultured with the solution of lipofectamine and MAPT siRNA complex for 48 hours. The protein concentration was measured by using the Bradford method.^14^ The protein samples (50 μg) were run through 12.5% sodium dodecyl sulfate–polyacrylamide gel electrophoresis.^14^ After the electrophoretic transfer of the protein to a nitrocellulose membrane, non‐specific binding was blocked by incubation with 5% skim milk in 20 m mol L^−1^ Tris‐HCl (pH=7.5, 0.5 M NaCl), or tris‐buffered saline (TBS), for 1 hour at room temperature.^14^ After being washed three times with TBS that contained 0.05% Tween 20 (TTBS), the blots were probed with a 1:200 dilution of rabbit polyclonal immunoglobulin (Ig)G that is specific for human MAPT (sc‐5587; Santa Cruz Biotechnology) for 2 hours.^14^ The blots were also probed with a 1:1000 dilution of monoclonal anti‐β‐actin antibody (Sigma‐Aldrich) in order to be relatively quantified. β‐Actin was used as a loading control.^14^ Then, the membranes were washed three times with TTBS and incubated for 1 hour at room temperature with an anti‐rabbit IgG horseradish peroxidase (HRP) ‐linked antibody (Cell Signaling Technology, Beverly, MA, USA) for MAPT and an anti‐mouse IgG HRP‐linked antibody (Cell Signaling Technology) for β‐actin, respectively.^14^ After being washed three times with TTBS, the membrane was transferred to enhanced chemiluminescence (ECL) Western blotting detection reagents (GE Healthcare, Buckinghamshire, UK) and incubated in this solution for 60 seconds at room temperature.^14^ The protein bands on the membrane were visualized by using ECL (ChemiDoc XRS; Biorad, Hercules, CA, USA), according to the manufacturer's instructions.^12^ The band intensity was analyzed with Molecular Imager, Image Lab v. 3.0.1 (Bio‐Rad).

### 2.7 Cell proliferation assay

The level of cell proliferation was assayed by using a cell‐counting kit (CCK‐8; Dojin Laboratories, Kumamoto, Japan).^12^ The TOV112D and HRA cells were cultured overnight in 96‐well microplates at 4.0×10^4^ cells per well with 100 μL of medium. Afterwards, a solution of lipofectamine and DNA complex that contained 0.1 μg of human MAPT DNA was added to the cells. The control group was given only a mixture of the vector and lipofectamine. The transfected cells were incubated at 37°C in a CO_2_ incubator for 48 hours.^12^ Afterwards, paclitaxel was added to the cells in four pattern concentrations—0, 1, 10, and 50 μg/mL—and incubated at 37°C in a CO_2_ incubator for 48 hours. The level of cell proliferation was assessed 4 hours after the addition of the CCK‐8 by measuring A_450_ with a Multiskan FC microplate reader (Thermo scientific, Yokohama, Japan).^12^ A preliminary study using this kit showed that the absorbance was directly proportional to the number of cells.^12^ The experiment was conducted in triplicate. The proliferation of the TOV112D and OVCAR3 cells also were assessed by adding a solution of lipofectamine and siRNA complex that contained 5 pmol human MAPT siRNA.

### 2.8 Cell cycle analysis

A cell cycle analysis was performed by using the Cell Cycle Phase Determination Kit (Cayman chemical, Ann Arbor, MI, USA). The TOV112D (control), MAPT DNA‐transfected TOV112D, and MAPT siRNA‐transfected TOV112D cells were collected and centrifuged to pellet them at the bottom. After centrifugation, the cells were washed twice with an assay buffer and the cell pellet was resuspended to a density of 10^6^ cells/mL in the assay buffer. In order to fix and permeabilize the cells, 1 mL of fixative was added to each sample. Next, the fixed cells were centrifuged at 500 *g* for 5 minutes and the fixative was decanted. The cell pellet was suspended in 0.5 mL of staining solution and incubated for 30 minutes at room temperature. The cell cycle was analyzed in the FL2 channel of a flow cytometer with a 450 nm excitation laser (Becton, Dicknson and Company, Franklin Lakes, NJ, USA).

### 2.9 Apoptosis analysis

The apoptosis analysis was performed by using the Annexin V‐FITC (fluorescein isothiocyanate) Apoptosis Detection Kit (Beckman Coulter, Brea, CA, USA). The TOV112D (control), MAPT DNA‐transfected TOV112D, and MAPT siRNA‐transfected TOV112D cells were collected and washed with PBS after centrifugation for 5 minutes at 500 *g* and at 4°C. The supernatant was discarded and the cell pellet was resuspended in the diluted binding buffer to 5×10^5^ cells/mL. Next, 5 μL of Annexin V‐FITC solution and 2.5 μL of dissolved propidium iodide were added to 100 μL of the cell suspension. The tubes were kept on ice and incubated for 10 minutes in the dark. Then, 400 μL of the binding buffer was added and the cell samples were analyzed by flow cytometry.

### 2.10 Statistical analysis

All the statistical analyses were performed by using the Student's *t* test and all the *P*‐values that were <.05 were considered to be statistically significant.

## 3 Results

### 3.1 Expression levels of the Tau protein in various cell lines

**Figure 1:**
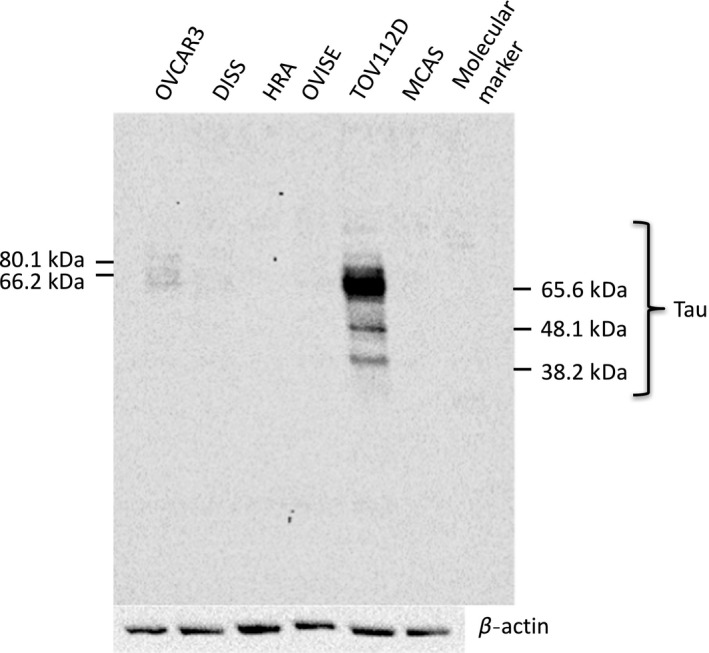
Western blot analysis for the Tau protein expression levels. The level of Tau protein expression was the strongest in the TOV112D cells. The microtubule‐associated protein Tau included six isoforms between 46‐80 kDa and its antibody detected multiple bands in the range

**Figure 2:**
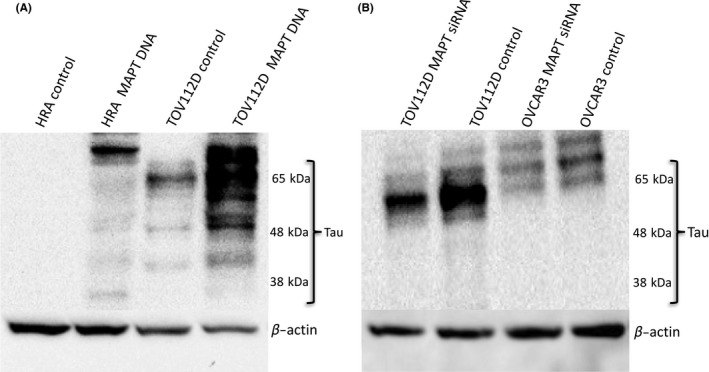
Alteration of Tau protein expression after microtubule‐associated protein Tau (MAPT) DNA or small interfering RNA (siRNA) transfection. A. The comparison of the level of Tau protein expression between the control cells and the MAPT DNA‐transfected cells on the Western blot analysis. The expression of the Tau protein increased by the transfection of the MAPT DNA in the TOV112D cells and the HRA cells. In particular, transfection of the MAPT DNA showed multiple bands and its phenomenon suggested hyperphosphorylation. B. The comparison of the level of Tau protein expression between the control cells and the MAPT siRNA‐transfected cells on the Western blot analysis. The expression of the Tau protein decreased by the transfection of the MAPT siRNA in the TOV112D cells and the OVCAR3 cells

A Western blot analysis showed that the expression level of the Tau protein was the highest in the TOV112D cells (Fig. 1), whereas it was expressed weakly in the OVCAR3 cells. In contrast, it was residually expressed in the other four cell lines. The MAPT included six isoforms between 46 and 80 kDa and its antibody detected multiple bands in the range. Figure 2A shows a comparison of the Tau protein expression in the TOV112D control cells and the MAPT DNA‐transfected TOV112D cells and in the HRA control cells and the MAPT DNA‐transfected HRA cells. A similar comparison was performed between the TOV112D control and the siRNA‐transfected TOV112D cells and the OVCAR3 control and the MAPT siRNA‐transfected OVCAR3 cells (Fig. 2B). In the TOV112D and HRA cells, transfection with MAPT DNA increased the expression of the Tau protein, whereas in the TOV112D and OVCAR3 cells, transfection with MAPT siRNA decreased the expression of the Tau protein (Fig. 2A,B). In particular, the transfection of MAPT DNA showed multiple bands and its phenomenon suggested hyperphosphorylation.^15^

### 3.2 Changes in cell proliferation after microtubule‐associated protein Tau DNA or microtubule‐associated protein Tau small interfering RNA transfection

**Figure 3:**
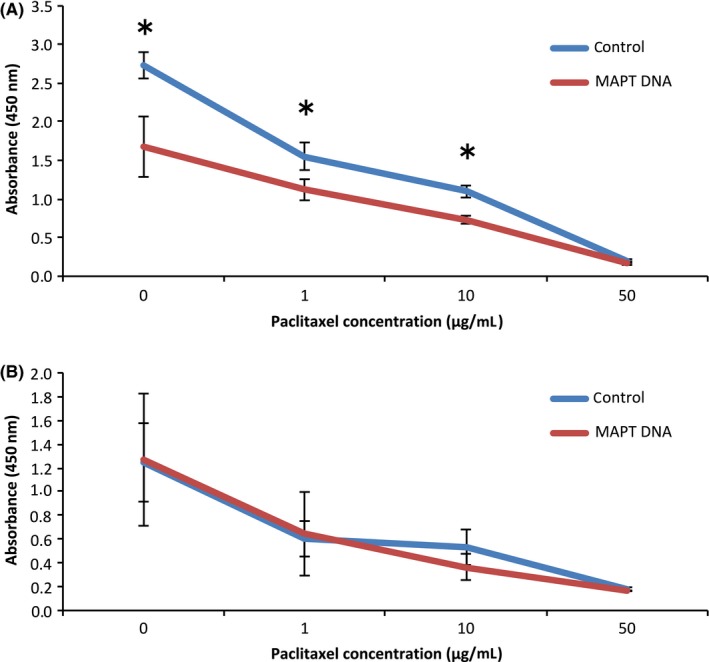
Cell proliferation assay. A. The cell proliferation of the TOV112D cells that were transfected with the microtubule‐associated protein Tau (MAPT) DNA was significantly more inhibited, compared with the controls, at 0, 1, and 10 μg/mL paclitaxel. *P<.05. B. The cell proliferation of the HRA cells that were transfected with the MAPT DNA was not inhibited, compared with the controls, in almost all concentrations of paclitaxel

**Figure 4:**
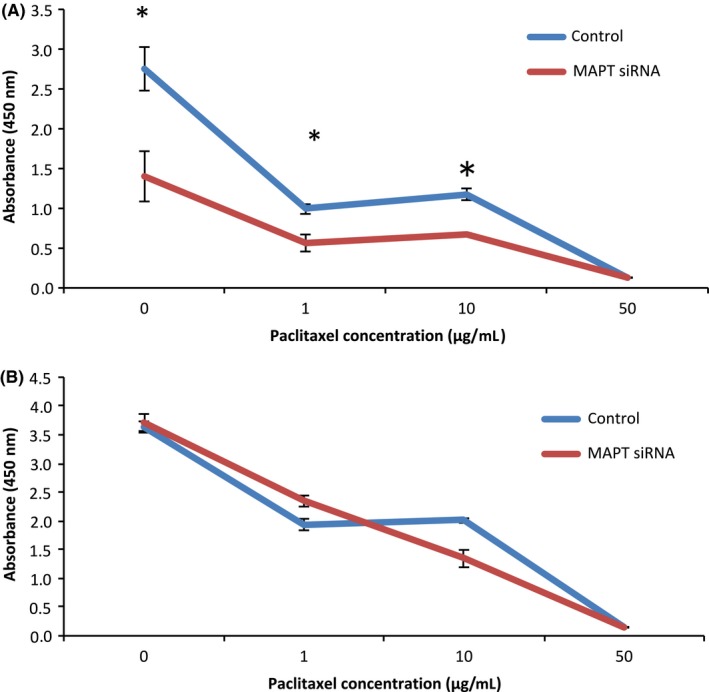
Cell proliferation assay. (A) The cell proliferation of the TOV112D cells that were transfected with the microtubule‐associated protein Tau (MAPT) small interfering RNA **(**siRNA) was significantly more inhibited, compared with the controls, at 0, 1, and 10 μg/mL paclitaxel. *P<.05. (B) The cell proliferation of the OVCAR‐3 cells that were transfected with the MAPT siRNA was not inhibited, compared with the controls, in almost all the concentrations of paclitaxel

Figure 3 shows the changes in cell proliferation, as assessed by measuring A_450_ with a microplate reader 4 hours after the addition of CCK8. The proliferation of the MAPT DNA‐transfected TOV112D cells was significantly more inhibited than that of the control cells at paclitaxel concentrations of 0, 1, and 10 μg/mL (Fig. 3A). The proliferation of the MAPT DNA‐transfected HRA cells was not inhibited, compared to that of the control cells, at almost all paclitaxel concentrations (Fig. 3B). The proliferation of the MAPT siRNA‐transfected TOV112D cells was significantly more inhibited than that of the control cells at paclitaxel concentrations of 0, 0.1, 1, and 10 μg/mL (Fig. 4A). The proliferation of the MAPT siRNA‐transfected OVCAR3 cells was not inhibited, compared to that of the control cells, at almost all paclitaxel concentrations (Fig. 4B). Note here that MAPT DNA transfection or MAPT siRNA transfection significantly inhibited the cell proliferation of TOV112D without the administration of paclitaxel.

### 3.3 Cell cycle changes after microtubule‐associated protein Tau DNA or microtubule‐associated protein Tau small interfering RNA transfection

**Figure 5:**
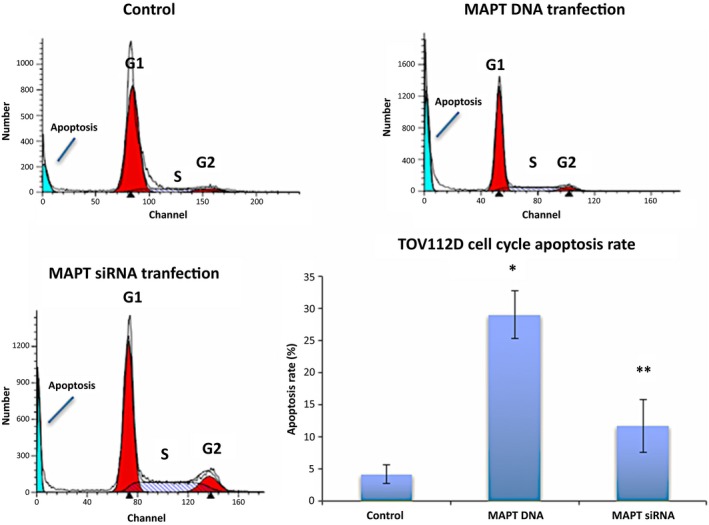
Cell cycle analysis. The blue areas in the left part of the figures show the subG1 phase showing apoptosis. The average apoptosis rate was 4.1% for the control cells and 29.0% for the DNA‐transfected cells. There was a significant increase in apoptosis of the DNA‐transfected cells. *P<.001, compared to the control. The average apoptosis rate was 11.7% for the small interfering RNA (siRNA)‐transfected cells. There was a significant increase in apoptosis of the microtubule‐associated protein Tau (MAPT) siRNA‐transfected cells. **P<.05, compared to the control

As MAPT DNA transfection or MAPT siRNA transfection altered the cell proliferation of TOV112D, the cell cycle in the TOV112D cells with their transfection was analyzed. The average apoptosis rate in the control cells was 4.1% and the average apoptosis rate of the cells that had been transfected with DNA and siRNA was 29.0% and 11.7%, respectively. The rate of apoptosis increased significantly in the cells that had been transfected with MAPT DNA or siRNA (*P*<.05, Fig. 5).

### 3.4 Alteration of apoptosis after microtubule‐associated protein Tau DNA or microtubule‐associated protein Tau small interfering RNA transfection

**Figure 6:**
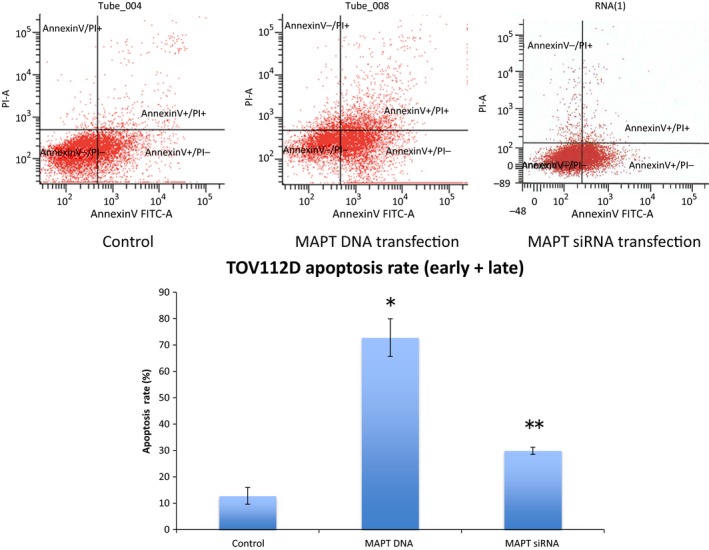
Apoptosis analysis. The Annexin‐V+/PI– cells show early apoptosis and the Annexin‐V+/PI+ cells show late apoptosis. The average total (early + late) apoptosis rate of the control cells was 12.8% (average early apoptosis, 11.3%; average late apoptosis, 1.5%). The average total apoptosis rate of the DNA‐transfected cells was 72.8% (average early apoptosis, 52.6%; average late apoptosis, 19.7%). There was a significant increase in apoptosis of the DNA‐transfected cells. *P<.0001, compared to the control. The average total apoptosis rate of the small interfering RNA (siRNA)‐transfected cells was 29.9% (average early apoptosis, 27.6%; average late apoptosis, 2.3%). There was a significant increase in apoptosis of the siRNA‐transfected‐cells. **P<.001, compared to the control. FITC **,** fluorescein isothiocyanate

Although the Annexin‐V+/PI– cells showed early apoptosis, the Annexin‐V+/PI+ cells showed late apoptosis. In the control cells, the average rate of total apoptosis was 12.8% (average early apoptosis, 11.3%; late apoptosis, 1.5%). In the DNA‐transfected cells, the average total was 72.8% (average early apoptosis rate, 52.6%; average late apoptosis, 19.7%). In the siRNA‐transfected cells, the average rate of total apoptosis was 29.9% (average early apoptosis, 27.6%; average late apoptosis, 2.3%). The rates of apoptosis increased significantly in the DNA‐transfected cells and in the siRNA‐transfected cells (*P*<.0001 and *P*<.001, respectively, Fig. 6).

## 4 Discussion

First, the level of the Tau protein expression in various OC cells was researched. As shown in Figure 1, the Tau protein expression level is different according to the cell type. The level of Tau expression was the highest in the TOV112D cells. These are a type of endometrioid carcinoma. Therefore, the Tau protein expression levels might be higher in endometrioid carcinoma than in other types of OC. OVCAR3, DISS, and HRA are types of serous carcinoma. OVISE is a type of clear cell carcinoma. MCAS is a type of mucinous carcinoma. In a previous study, the primary tumors of 74 patients with OC were immunohistochemically stained for the Tau protein.^4^ The number of Tau‐positive patients was compared between the patients with the serous and those with the non‐serous type. However, it was reported that there was no significant difference between the histological types. In this study, there was a difference in the Tau expression levels among patients with the same histological type. The HRA and OVCAR3 cells are types of serous carcinoma and there are many other histological types of OC cells. Accordingly, there is the need to research Tau protein level expression in other cell types. One study compared the level of Tau protein expression among five types of serous OC cells.^16^ The level of Tau protein expression was different in each type.^16^ The reason for the difference in Tau protein expression among the histological types or among individuals is unclear.^16^ Other research reported that repeated sequences, CpG islands, and haplotypes affect the DNA and RNA levels of MAPT and regulate MAPT gene expression.^17^ These factors might have different actions among the cells of each histological type.

The Tau protein recently has been identified as a marker of response to paclitaxel in breast cancer.^5^, ^18^, ^19^, ^20^, ^21^, ^22^ The Tau protein and paclitaxel compete to bind to the microtubules. Therefore, the sensitivity to paclitaxel should be weak in cells with a high Tau protein expression. A study examined the Tau expression of 74 patients with epithelial OC and compared the progression‐free survival (PFS) and 3 year overall survival (OS).^4^ The PFS of the Tau‐negative patients was significantly better than that of the Tau‐positive patients (*P*=.0355). In the univariate analysis, the 3 year OS of the Tau‐negative patients was significantly better than that of the Tau‐positive patients (*P*=.0198).^4^ However, in the multivariate analysis, there was no significant difference.^4^ It was concluded that negative Tau protein expression seems to be both a good prognostic factor and a predictor of the response to paclitaxel and platinum‐based chemotherapy in patients with epithelial OC.^4^ Conversely, another study reported that Tau expression was not associated with PFS or OS in serous epithelial OC.^23^ Furthermore, many other factors affect PFS and OS, including the histological type, stage, residual tumor size, and age. Chemosensitivity is also influenced by many factors.^24^, ^25^, ^26^, ^27^, ^28^ In the patients with epithelial OC, a sensitivity to both paclitaxel and platinum affected their response to chemotherapy. Therefore, the current authors investigated the relationship between paclitaxel use and Tau protein expression in the epithelial OC cells. It was expected that the regulation of cell proliferation by paclitaxel would become stronger by transfection of the MAPT siRNA (Tau down‐regulation). It also was expected that the regulation of cell proliferation by paclitaxel would become weaker by transfection of the MAPT DNA. As shown in Figure 4, in the TOV112D cells, cell proliferation was more inhibited by transfection of the MAPT siRNA. The authors specifically noted that cell proliferation was significantly inhibited in the cells with MAPT siRNA transfection without paclitaxel. The transfection of the MAPT siRNA resulted in the regulation of cell proliferation. In the OVCAR3 cells, similar changes were not observed because the level of Tau protein expression in the OVCAR3 cells originally was low. Among the TOV112D cells, the cell proliferation of the MAPT DNA‐transfected cells also was more inhibited without paclitaxel (Fig. 3). Thus, the down‐regulation and overexpression of Tau protein regulate cell proliferation. In the HRA cells with low levels of Tau protein expression, similar changes were not observed. It had been anticipated that the down‐regulation of the Tau protein would cause inhibition of the polymerization of the microtubules. It also was anticipated that the overexpression of the Tau protein would cause inhibition of the depolymerization of the microtubules. Both these processes lead to apoptosis of the cells. Figure 6 shows the increase in cell apoptosis of the TOV112D cells that were transfected with MAPT DNA and siRNA. The level of apoptosis increased in both the MAPT DNA‐transfected cells and the MAPT siRNA‐transfected cells, but the ratio of late apoptosis to total apoptosis increased in the MAPT DNA‐transfected cells. This could be because the mechanism of cell apoptosis is different between the MAPT DNA‐transfected cells and the MAPT siRNA‐transfected cells. It is speculated that the cytostatic effect by transfection of MAPT DNA is more rapid and stronger, compared with that by transfection of MAPT siRNA. The hyperphosphorylation of MAPT might deprive it of its binding ability to microtubules and cause apoptosis.^1^ In fact, the MAPT DNA transfection led to the hyperphosphorylation of MAPT, as shown in Figure 2A. However, the relevance of the increase in the ratio of late apoptosis is unclear.

The inhibition of cell proliferation by the transfection of MAPT DNA or siRNA was observed only in the TOV112D cells. The reason for this inhibition could be the high level of Tau protein expression in the TOV112D cells. The extent of contribution of the Tau protein in cell proliferation might differ in each cell line. The limitation of this study was that such a phenomenon was demonstrated just in the TOV112D cells. In order to indicate a possibility of new molecularly targeted therapy in Tau‐positive cells, further studies should confirm the cytostatic effect by the down‐regulation and overexpression of Tau proteins by using other cell lines, such as for breast cancer and neurological malignancy, with high Tau protein expression. The details of the apoptosis mechanism also need to be elucidated. An obvious limitation of this study is that there was no examination of clinical samples. As the phenotype of the cell lines does not always reflect their original characteristics, future studies should show a relationship between apoptosis and MAPT distribution and expression in the clinical samples of OC. If this is done, the cytostatic effect by the down‐regulation and overexpression of Tau protein can be analyzed in vivo.

## Disclosure

*Conflict of interest*: The authors declare no conflict of interest. All the procedures that were followed were in accordance with the ethical standards of the responsible committees on human experimentation (institutional and national). *Animal rights*: This article does not contain any study with animal participants that was performed by any of the authors.

## References

[1] Komuro Y , Xu G , Bhaskar K , Lamb BT . Human tau expression reduces adult neurogenesis in a mouse model of tauopathy. Neurobiol Aging. 2015;36:2034–2042. 25863528 10.1016/j.neurobiolaging.2015.03.002 PMC4724414

[2] Mohan R , John A . Microtubule‐associated proteins as direct crosslinkers of actin filaments and microtubules. IUBMB Life. 2015;67:395–403. 26104829 10.1002/iub.1384

[3] Kavallaris M , Kuo DY , Burkhart CA , et al. Taxol‐resistant epithelial ovarian tumors are associated with altered expression of specific beta‐tubulin isotypes. J Clin Invest. 1997;100:1282–1293. 9276747 10.1172/JCI119642 PMC508306

[4] Smoter M , Bodner L , Grala B , et al. Tau protein as a potential predictive marker in epithelial ovarian cancer patients treated with paclitaxel/platinum first‐line chemotherapy. J Exp Clin Cancer Res. 2013;32:25. 23631819 10.1186/1756-9966-32-25 PMC3654950

[5] Rouzier R , Rajan R , Wagner P , et al. Microtubule‐associated protein Tau: a marker of paclitaxel sensitivity in breast cancer. Proc Natl Acad Sci USA. 2005;102:8315–8320. 15914550 10.1073/pnas.0408974102 PMC1149405

[6] Manning AP , Mes‐Masson AM , Seymour RJ , Tetrault M , Provencher DM , Tonin PN . Expression of FHIT in primary cultures of human epithelial ovarian tumors and malignant ovarian ascites. Mol Carcinog. 1999;24:218–225. 10204806 10.1002/(sici)1098-2744(199903)24:3<218::aid-mc8>3.0.co;2-a

[7] Kidera Y , Yoshimura T , Ohkuma Y , Iwasaka T , Sugimori H . [Establishment and characterization of a cell line derived from mucinous cystadenocarcinoma of human ovary.] Nihon Sanka Fujinka Gakkai Zasshi 1985;37:1820–1824 (in Japanese with English abstract). 4056530

[8] Hamilton TC , Young RC , McKoy WM , et al. Characterization of a human ovarian carcinoma cell line (NIH:OVCAR‐3) with androgen and estrogen receptors. Cancer Res. 1983;43:5379–5389. 6604576

[9] Gorai I , Nakazawa T , Miyagi E , Hirahara F , Nagashima Y , Minaguchi H . Establishment and characterization of two human ovarian clear cell adenocarcinoma lines from metastatic lesions with different properties. Gynecol Oncol. 1995;57:33–46. 7535723 10.1006/gyno.1995.1097

[10] Kikuchi Y , Kizawa I , Oomori K , et al. Establishment of a human ovarian cancer cell line capable of forming ascites in nude mice and effects of tranexamic acid on cell proliferation and ascites formation. Cancer Res. 1987;47:592–596. 3791243

[11] Yokoyama Y , Xin B , Shigeto T , et al. Clofibric acid, a peroxisome proliferator‐activated receptor α ligand, inhibits growth of human ovarian cancer. Mol Cancer Ther. 2007;6:1379–1386. 17431116 10.1158/1535-7163.MCT-06-0722

[12] Kobayashi A , Yokoyama Y , Osawa Y , Miura R , Mizunuma H . Gene therapy for ovarian cancer using carbonyl reductase 1 DNA with a polyamidoamine dendrimer in mouse models. Cancer Gene Ther. 2016;23:24–28. 26584532 10.1038/cgt.2015.61

[13] Osawa Y , Yokoyama Y , Shigeto T , Futagami M , Mizunuma H . Decreased expression of carbonyl reductase 1 promotes ovarian cancer growth and proliferation. Int J Oncol. 2015;46:1252–1258. 25572536 10.3892/ijo.2014.2810

[14] Sakamoto A , Yokoyama Y , Umemoto M , et al. Clinical implication of expression of cyclooxygenase‐2 and peroxisome proliferator activated‐receptor gamma in epithelial ovarian tumours. Br J Cancer. 2004;91:633–638. 15266333 10.1038/sj.bjc.6602009 PMC2364772

[15] Feijoo C , Campbell DG , Jakes R , Goedert M , Cuenda A . Evidence that phosphorylation of the microtubule‐associated protein Tau by SAPK4/p38delta at Thr50 promotes microtubule assembly. J Cell Sci. 2005;118:397–408. 15632108 10.1242/jcs.01655

[16] Gurler H , Yu Y , Choi J , Kajdacsy‐Balla AA , Berbolina MV . Three‐dimensional collagen type I matrix up‐regulates nuclear isoforms of the microtubule associated protein Tau implicated in resistance to paclitaxel therapy in ovarian carcinoma. Int J Mol Sci. 2015;16:3419–3433. 25658796 10.3390/ijms16023419 PMC4346904

[17] Caillet‐Boudin ML , Buée L , Sergeant N , Lefebvre B . Regulation of human MAPT gene expression. Mol Neurodegener. 2015;10:28. 26170022 10.1186/s13024-015-0025-8 PMC4499907

[18] Koo DH , Lee HJ , Ahn JH , et al. Tau and PTEN status as predictive markers for response to trastuzumab and paclitaxel in patients with HER2‐positive breast cancer. Tumour Biol. 2015;36:5865–5871. 25725586 10.1007/s13277-015-3258-9

[19] Zhou J , Qian S , Li H , et al. Predictive value of microtubule‐associated protein Tau in patients with recurrent and metastatic breast cancer treated with taxane‐containing palliative chemotherapy. Tumour Biol. 2015;36:3941–3947. 25773385 10.1007/s13277-015-3037-7

[20] Bonneau C , Gurard‐Levin ZA , Andre F , Pusztai L , Rouzier R . Predictive and prognostic value of the Tau protein in breast cancer. Anticancer Res. 2015;35:5179–5184. 26408675

[21] Li ZH , Xiong QY , Tu JH , et al. Tau proteins expressions in advanced breast cancer and its significance in taxane‐containing neoadjuvant chemotherapy. Med Oncol. 2013;30:591. 23681778 10.1007/s12032-013-0591-y

[22] Wang K , Deng QT , Liao N , et al. Tau expression correlated with breast cancer sensitivity to taxanes‐based neoadjuvant chemotherapy. Tumour Biol. 2013;34:33–38. 22976542 10.1007/s13277-012-0507-z

[23] Steffensen KD , Smoter M , Waldstrøm M , et al. Resistance to first line platinum paclitaxel chemotherapy in serous epithelial ovarian cancer: the prediction value of ERCC1 and Tau expression. Int J Oncol. 2014;44:1736–1744. 24585004 10.3892/ijo.2014.2311

[24] Böhm S , Faruqi A , Said I , et al. Chemotherapy Response Score: development and validation of a system to quantify histopathologic response to neoadjuvant chemotherapy in tubo‐ovarian high‐grade serous carcinoma. J Clin Oncol. 2015;33:2457–2463. 26124480 10.1200/JCO.2014.60.5212

[25] Sehouli J , Fotopoulou C , Erol E , et al. Alopecia as surrogate marker for chemotherapy response in patients with primary epithelial ovarian cancer: a metaanalysis of four prospective randomised phase III trials with 5114 patients. Eur J Cancer. 2015;51:825–832. 25771433 10.1016/j.ejca.2015.01.008

[26] Chen S , Jiao JW , Sun KX , Zong ZH , Zhao Y . MicroRNA‐133b targets glutathione S‐transferase π expression to increase ovarian cancer cell sensitivity to chemotherapy drugs. Drug Des Devel Ther. 2015;9:5225–5235. 10.2147/DDDT.S87526 PMC4577257 26396496

[27] Zhang Y , Yu JJ , Tian Y , et al. eIF3a improve cisplatin sensitivity in ovarian cancer by regulating XPC and p27Kip1 translation. Oncotarget. 2015;6:25441–25451. 26213845 10.18632/oncotarget.4555 PMC4694843

[28] Zhang GN , Liu H , Huang JM , et al. TP53 K351N mutation‐associated platinum resistance after neoadjuvant chemotherapy in patients with advanced ovarian cancer. Gynecol Oncol. 2014;132:752–757. 24463159 10.1016/j.ygyno.2014.01.028

